# Enhancing the Predictive Value of Formative Evaluation in Extended Reality Adoption: Addressing the Experience Gap

**DOI:** 10.2196/93029

**Published:** 2026-04-22

**Authors:** José Ferrer Costa, Manuel Armayones Ruiz

**Affiliations:** 1 Research and Innovation, Badalona Serveis Assistencials, Pl Pau Casals 1, Badalona, Barcelona, 08911, Spain, +34937407482; 2 Grup de Recerca Multidisciplinar en Salut i Societat (GREMSAS), IDIAP Jordi Gol, Institut Universitari d'Investigació en Atenció Primària Jordi Gol, Barcelona, Catalonia, Spain; 3 Behavioural Design Lab (BDLab), UOC eHealth Centre, Faculty of Psychology and Education Sciences, Universitat Oberta de Catalunya, Barcelona, Catalonia, Spain

**Keywords:** extended reality, formative evaluation, implementation science, experience gap, perception-based evaluation, embodied interaction, digital health

## Abstract

Formative evaluation is widely used in implementation science to anticipate barriers and facilitators prior to the deployment of health technologies, typically relying on stakeholders’ reported beliefs collected before real-world exposure. This approach has proven informative for many digital health tools; however, its application to immersive and embodied technologies such as extended reality (XR) warrants closer scrutiny. Most XR interventions in health care are delivered through head-mounted displays, which depend on spatial perception and sensorimotor engagement. Several implementation-relevant properties, including comfort, perceived intrusiveness, safety, and workflow disruption, often become apparent only through direct interaction. At the same time, large segments of the health care workforce remain XR-naive, such that preuse judgments are frequently shaped by anticipation rather than experience. Drawing on concepts from implementation science, grounded cognition, and human-computer interaction, this Viewpoint examines a plausible interpretive problem in XR adoption and argues that perception-based formative evaluation, when applied through frameworks developed for screen-based technologies, may misclassify barriers and facilitators. Rather than questioning formative evaluation as a methodological approach, we identify a boundary condition for its interpretability in experience-dependent technologies and propose a pragmatic refinement: incorporating brief experiential familiarization before eliciting stakeholder perceptions to strengthen early-stage assessment and improve alignment with real-world implementation decisions.

## Introduction

Formative evaluation is a core component of implementation science, intended to surface early barriers and facilitators that may influence the uptake of new health technologies [1]. This emphasis on early-stage evaluation aligns with international health policy priorities concerning system performance, resilience, and equitable access [2,3].

In most adoption and implementation models, preuse assessment relies on stakeholders’ expectations and perceived attributes to judge feasibility, readiness, and priority [4-6]. As health systems increasingly consider immersive technologies within broader processes of digital transformation, the adequacy of existing formative evaluation approaches becomes a matter of practical and policy relevance [7].

Extended reality (XR), including virtual, augmented, and mixed reality, differs from many conventional digital tools in that interaction unfolds through perceptually immersive environments and embodied engagement rather than indirect, screen-based interaction [8,9]. In health care, these systems are typically delivered through head-mounted displays in real clinical environments, often under professional supervision [10,11]. Theoretical and experimental work suggests that experiential phenomena such as presence and embodiment can shape attention, emotional response, and bodily perception in ways that are directly implicated in therapeutic effect [8,12,13]. Therefore, these characteristics are not incidental features of user experience but are part of the mechanism through which many XR interventions exert their effects.

These immersive systems introduce perceptual, cognitive, and sensory demands that are difficult to infer from description alone. Physical comfort, perceptual effort, situational awareness, attentional load, interface complexity, and sensory mismatch may influence tolerability, task performance, and learning; however, these properties often become apparent only through direct interaction [10,11,14,15]. This creates a methodological challenge for formative evaluation. Established adoption and implementation frameworks provide validated constructs for assessing anticipated value and feasibility [1,4,5]; however, they say little about how such perceptions should be interpreted when a technology’s defining features only emerge through embodied interaction. For experience-dependent systems such as XR, clarifying the scope and limits of early stakeholder judgments becomes important [1,10].

This paper does not report original comparative data on pre-exposure vs postexposure judgments. Rather, it offers a conceptual and methodological analysis that draws on literature from implementation science, grounded cognition, human-computer interaction, and XR adoption research. Its purpose is to clarify a plausible interpretive problem in formative evaluation, define the experience gap as a hypothesis-generating construct, and propose brief experiential familiarization as a pragmatic preparatory step before eliciting selected stakeholder judgments. The literature cited was selected to reflect complementary perspectives on how technologies are appraised before use, how experiential properties emerge through interaction, and how user experience may shape adoption and evaluation.

## Limits of Perception-Based Evaluation in XR Adoption

Formative evaluation often assumes that stakeholders can anticipate barriers and facilitators before deployment. This is more plausible when the technology resembles tools already embedded in routine practice, as respondents can draw on prior experience to ground their judgments [4,5]. With immersive technologies such as XR, that experiential grounding is often absent.

At the point when formative assessments are typically conducted, direct clinical experience with XR remains absent or limited among health care professionals. Survey data support this pattern. In a public hospital study, only 5% of clinicians reported having used virtual reality (VR) in clinical care [16]. Similarly, a large survey of physical therapy clinicians in the United States found that just 7.1% were currently using VR in practice [17]. In this context, responses to preuse questionnaires are predominantly expectation driven. Judgments about ease of use, intrusiveness, safety, or workflow compatibility are formed without an experiential reference and may reflect imagined difficulty or general uncertainty rather than properties encountered during actual use.

Qualitative research echoes this pattern. Users with no prior XR experience may have limited ability to anticipate what interaction will involve, sometimes stating explicitly that, beforehand, they did not know what to expect prior to immersion [18]. In these circumstances, early perceptions may be recorded as barriers even when they reflect uncertainty rather than direct experience. This is particularly evident when unfamiliarity with immersive systems is combined with blurred distinctions between VR, augmented reality, and conventional screen-based technologies [10]. Therefore, apparent barriers may partly reflect concerns about the modality being judged rather than stable evidence of its usability.

Theoretical work in grounded cognition helps explain why these difficulties arise. When prior sensorimotor experience is absent, people rely on partial mental simulation to evaluate tools and actions, which limits the accuracy of anticipatory judgments [19]. Similar concerns have long been noted in human-computer interaction research, where usability and interaction-related properties are recognized as hard to assess without hands-on engagement, especially for systems that depend on embodied interaction [20].

These constraints are visible in implementation-oriented XR research. Consensus exercises and survey-based studies often ask stakeholders to judge feasibility, barriers, and facilitators despite heterogeneous or minimal direct exposure to XR systems [16,21,22]. The resulting assessments frequently combine expressed interest with uncertainty about practical implications. By contrast, qualitative studies suggest that even limited experiential exposure can shift evaluations away from abstract concerns toward more concrete appraisals of clinical value and implementation requirements [23], while implementation reviews indicate that many early assessments are based on expected rather than observed factors [10].

This body of evidence suggests that preuse perceptions in XR-naive samples are shaped less by experience-informed judgment than by incomplete understanding of the modality being assessed. In the context of immersive technologies, this makes formative findings harder to interpret and increases the risk that early barriers and facilitators reflect expectations and concerns more than observed use or hands-on experience.

## The Experience Gap and the Misclassification of Adoption Barriers

**Figure 1.**
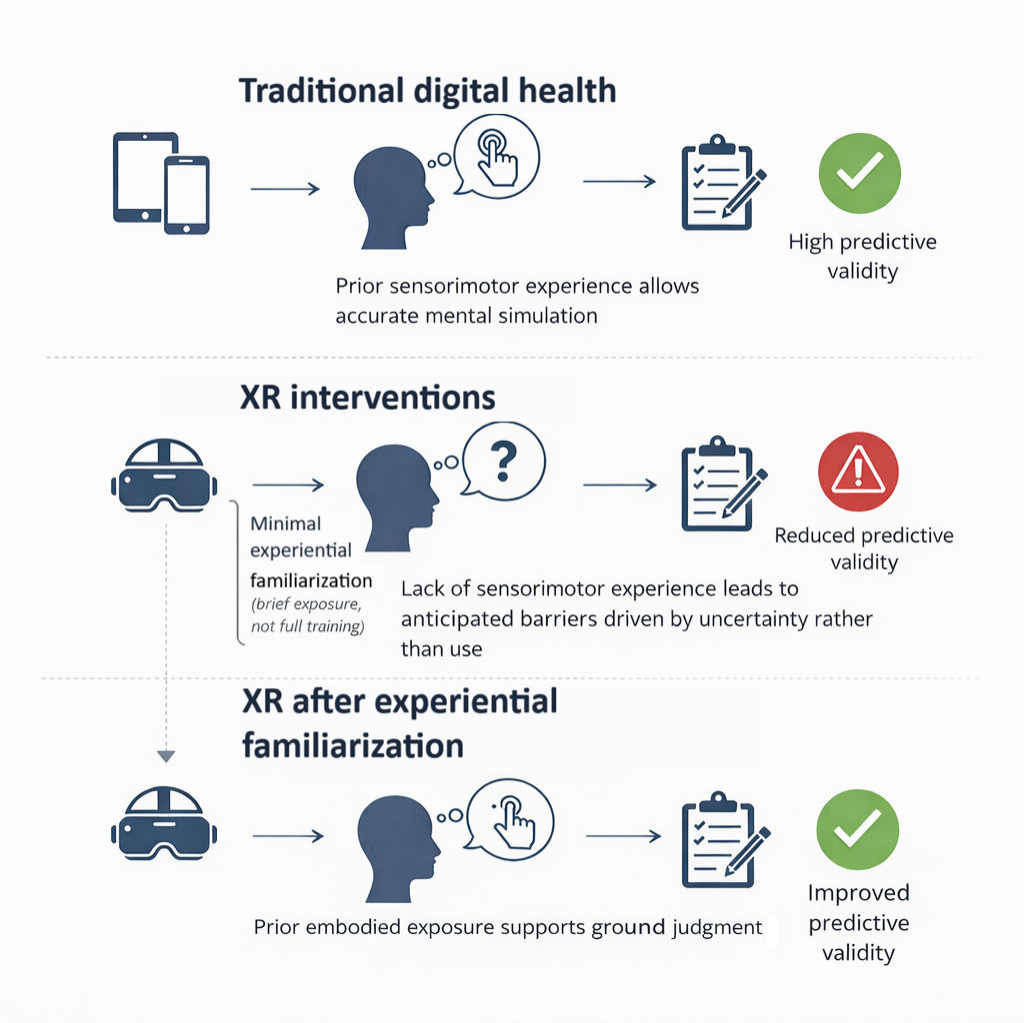
Conceptual model of the “experience gap” in perception-based formative evaluation, proposed here as a hypothesis-generating framework. For conventional digital health tools, stakeholders often have sufficient prior interaction experience to support plausible anticipatory judgments. In extended reality (XR), limited sensorimotor exposure reduces the interpretability of preuse assessments and raises the risk that uncertainty is recorded as an implementation barrier. Experiential familiarization is proposed as a pragmatic methodological condition to establish a minimal reference point before eliciting feasibility and acceptability judgments, rather than as a validated or sufficient intervention.

The limited experiential grounding described previously leads to a specific problem for perception-based formative evaluation in XR adoption. When stakeholder judgments are collected before any direct interaction with the technology, commonly used indicators of feasibility and readiness may no longer behave as stable signals of likely future use. Rather, they may reflect judgments formed under a partial understanding of what the technology actually entails. We refer to this interpretive mismatch as the experience gap. Figure 1 presents a conceptual model illustrating the hypothesized relationship between experiential exposure and perception-based evaluation and is intended as a hypothesis-generating framework rather than an empirically validated representation.

In this situation, familiar formative constructs used to characterize feasibility and readiness may no longer capture reflections grounded in prior practice. Rather, they express attempts to reason forward in the absence of concrete experience. While these constructs are well established for technologies that resemble existing systems [4,5], their interpretability may be more limited when applied to immersive interventions, whose defining features are difficult to anticipate prior to use.

Therefore, in XR-naive contexts, preuse ratings may be more likely to signal hesitation, risk sensitivity, limited experiential grounding, or initial impressions formed before meaningful use rather than fully informed judgments about likely barriers or facilitators [18,23]. Under these conditions, early assessments may reflect assumptions that have not yet been grounded in direct experience with the technology, whether expressed as concern or as early enthusiasm.

This does not suggest abandoning formative evaluation in XR research, nor does it imply that stakeholder perceptions are uninformative. Rather, it points to a boundary for interpretation and an opportunity for improvement. When assessments are conducted entirely before any immersive exposure, their outputs should not be treated as definitive classifications of implementation barriers. Ignoring the experiential constraints under which these judgments are produced risks treating uncertainty-driven concerns as intrinsic properties of the technology or the clinical context, rather than as artifacts of when and how the evaluation was conducted.

## Epistemic and Structural Barriers

When formative evaluation takes place before any immersive exposure, certain concerns might be confounded with fixed obstacles to implementation. In XR research, this tendency may be less a sign of resistance than a consequence of how judgments are formed. At this point, it becomes useful to distinguish between epistemic uncertainty and structural barriers to implementation. Current formative frameworks do not always make this separation explicit, as they often assume that relevant perceptions can be elicited before sustained use [1,24,25].

Certain concerns seem to arise mainly because stakeholders have not yet had direct experience with XR and may therefore be assessing it under conditions of uncertainty [18,26]. These may relate to safety and comfort, including intrusiveness, discomfort, or cybersickness, as well as usability, acceptability, and the perceived value of immersive interaction [8,18]. Because such dimensions depend heavily on actual use, they are especially vulnerable to misclassification when judged before exposure [8,26]. In practice, this may give disproportionate weight to anticipated burden while leaving the experiential value of XR less clearly defined before first use.

By contrast, other barriers are likely to remain relevant regardless of prior immersive use. These include workflow integration, staffing, physical space, hardware logistics, infection control, technical support, and broader organizational conditions [10]. The methodological problem described here does not imply that all early concerns are misplaced. Rather, it suggests that this interpretive distortion may arise when epistemic uncertainty, together with the low confidence that often accompanies it, is interpreted and documented as though it reflected a stable structural barrier to implementation [1,10,18]. Current formative approaches do not always distinguish clearly between concerns that may diminish with exposure and barriers that remain structurally relevant [10,23].

These distinctions matter because formative evaluation findings rarely remain neutral observations. Early barrier profiles are often used to inform decisions about organizational readiness, resource allocation, and the continuation or discontinuation of pilot initiatives [1]. When judgments formed under conditions of uncertainty are documented as definitive barriers, they can influence strategic decisions about XR adoption independently of how the technology performs once introduced into practice [10]. The issue is not that formative evaluation lacks value, but that the timing and experiential context of data collection shape which barriers are identified and how firmly they are interpreted.

## Toward Experience-Informed Formative Evaluation

These distinctions have practical implications for how formative evaluation is conducted in XR. The refinement proposed here is to introduce brief experiential familiarization before stakeholders are asked to assess selected dimensions of feasibility, acceptability, or fit with practice. For such judgments to remain interpretable, stakeholder perceptions need to be elicited under conditions that provide at least minimal experiential grounding. This proposal differs both conceptually and practically from staged pilot testing. Although pilot implementation evaluates feasibility, workflow integration, and clinical use over time during early deployment, experiential familiarization functions as a limited preparatory step at the formative stage. Its primary purpose is to establish a shared experiential baseline before more formal implementation or procurement decisions are made.

At a practical level, such encounters can be brief and structured. A familiarization session may include 3 simple elements: initial operational handling of the equipment, a short first-person immersive experience, and an immediate postexposure assessment. This may take the form of a guided interaction lasting approximately 10 to 20 minutes, during which stakeholders handle the equipment, wear the head-mounted display, and complete simple tasks relevant to the intended use context, ideally using the same content or application under consideration for routine practice. Depending on logistical constraints, these sessions may be conducted individually or in small groups.

This approach is already used by the authors as a routine practice when introducing XR into new clinical settings and among teams considering its adoption. In these sessions, the familiarization process is typically projected onto an external screen so that participants can follow each step of the setup, from defining the safe interaction area and floor level to launching the selected application and beginning first-person use. This makes the operational demands of XR visible from the outset while allowing participants to observe and discuss the process collectively before or during direct interaction.

This brief exposure serves several complementary functions. First, practical interaction with the hardware and interface can provide an initial understanding of setup demands, coordination needs, and operational complexity [11]. Second, first-person use allows stakeholders to experience immersion, physical comfort, and attentional demands directly, helping to anchor their judgments more firmly in observed interaction than in abstract expectation [26]. It may also help temper initial enthusiasm by grounding expectations in concrete clinical tasks and operational demands rather than idealized assumptions [27].

**Figure 2.**
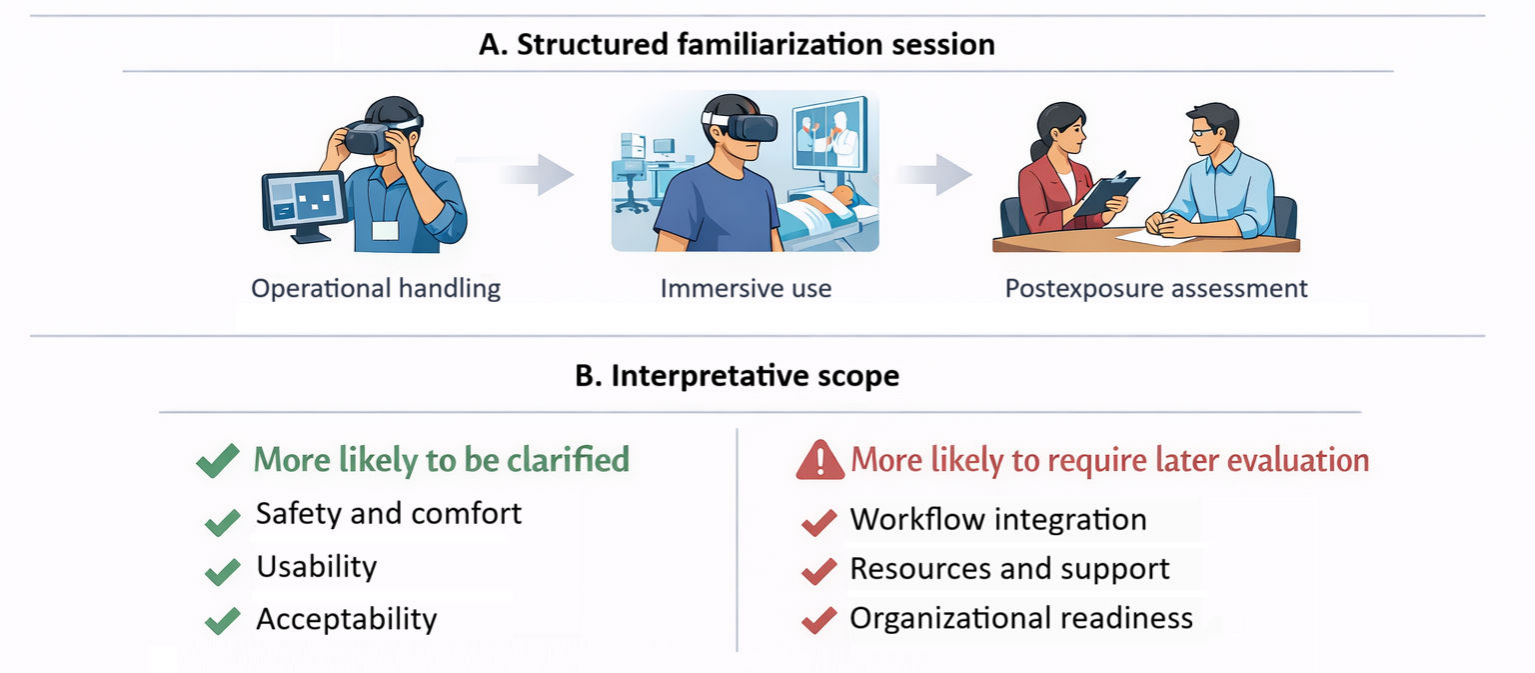
Proposed structure and interpretive scope of brief experiential familiarization in extended reality (XR) formative evaluation. (A) A brief structured familiarization session comprising operational handling, first-person immersive use, and postexposure assessment; and (B) evaluation domains, classified into those more likely to be clarified through brief familiarization, such as safety, comfort, usability, and acceptability, from broader implementation dimensions more likely to require later-stage evaluation, such as workflow integration, resources, support, and organizational readiness.

Several dimensions that are central to XR adoption are difficult to judge meaningfully without direct interaction. When sensorimotor experience is absent, anticipatory assessments rely on an incomplete mental simulation or expectations, constraining their accuracy [19,23]. Brief experiential familiarization does not eliminate uncertainty; however, it may allow it to be expressed in relation to experience rather than hypothetical assumptions [18,23]. This is particularly relevant for concerns that are experience dependent. By contrast, short-term encounters are not sufficient to assess broader implementation challenges or issues that emerge only through sustained use, such as technical and logistical problems or the organizational burden of integration across routine care pathways [10,11,28]. Figure 2 summarizes the proposed structure of this brief familiarization process and distinguishes the dimensions more likely to be clarified at this stage from those that typically require later implementation assessment.

At present, no single framework currently defines how a postdemo appraisal for XR should be structured, and the implementation literature points to a recurrent set of domains that are relevant to early decision-making. Reviews and consensus work have consistently identified usability, safety and comfort, workflow fit, training needs, resource demands, and organizational support as central determinants of XR adoption in health care, while broader implementation models remain useful for interpreting feasibility, readiness, and perceived value at the service level [10,11,21,24,25]. XR-specific guidance has also strengthened the reporting of safety and technical characteristics but has not yet established a practical structure for early appraisal across experiential, organizational, and strategic dimensions [29]. Therefore, in practice, an immediate postdemo assessment may reasonably address domains such as perceived clinical relevance, safety and comfort, usability, acceptability, workflow implications, training requirements, resource considerations, and perceived organizational fit, while recognizing that wider regulatory, ethical, and implementation issues usually require later-stage evaluation in real clinical settings.

A further implication concerns study design. Future research could make experiential conditions explicit within formative designs. For instance, pre-post approaches could compare stakeholders who receive brief, guided XR exposure with those who receive only informational or vicarious familiarization, such as written descriptions or nonimmersive materials. Examining within-person changes in perceived barriers and facilitators following hands-on interaction would allow researchers to assess whether preuse profiles among XR-naive stakeholders reflect imagined frictions more than judgments grounded in direct experience, as suggested by recent formative XR studies [23].

Importantly, this proposed approach remains compatible with existing implementation frameworks. It does not require new constructs or additional instruments. Established measures of acceptability, usability, and perceived usefulness continue to apply; however, their interpretive strength increases when responses are shaped by experience rather than anticipation alone. Framed in this way, brief experiential familiarization represents a modest refinement of current practice, improving the interpretability of early-stage findings while remaining aligned with the practical constraints of implementation research in immersive health technologies.

Beyond XR, similar interpretive limits may also affect other digital health technologies when their practical demands cannot be judged adequately without direct use, including applications such as artificial intelligence [30]. The issue may be particularly marked in XR, as perceptual immersion, sensorimotor interaction, and embodied experience are integral to usability, comfort, and perceived value [19,20].

## Conclusions

The difficulties examined in this viewpoint do not arise from inherent limitations of XR as a clinical technology. They reflect a methodological misalignment between the embodied character of immersive systems and the conditions under which formative evaluation is often conducted. When XR interventions are assessed primarily through preuse perceptions, particularly among stakeholders with limited prior exposure, judgments about feasibility and acceptability may be shaped by anticipation rather than by experience. In these circumstances, early findings risk being treated as stable indicators, even though they are better understood as provisional assessments formed under uncertainty.

This analysis does not diminish the role of formative evaluation in implementation science. Rather, it clarifies a boundary condition for its interpretation in experience-dependent technologies. For XR, the timing and experiential context of perception-based assessment are consequential. Introducing minimal embodied exposure before eliciting stakeholder judgments represents a pragmatic refinement that strengthens existing evaluative practice without requiring new frameworks, constructs, or measurement tools.

The contribution of this viewpoint is twofold. Conceptually, it frames the experience gap as a structural feature of XR adoption research, distinct from professional resistance, negative attitudes, or individual skill deficits. Methodologically, it proposes brief experiential familiarization as a low-burden preparatory step that may improve the interpretability of selected formative judgments before more formal implementation decisions are made.

For researchers, this perspective supports a more cautious reading of preuse perceptions in XR studies and closer alignment between formative designs and the embodied properties of immersive systems. For health systems and decision-makers, it underscores the value of experience-informed assessment when judging feasibility, acceptability, and readiness for implementation. More broadly, it suggests that some barriers reported in early XR research may reflect the timing and conditions of evaluation rather than obstacles that persist once technologies are encountered in practice. Brief experiential familiarization may help ensure that early judgments about XR reflect firsthand interaction more than unfamiliarity, making formative evaluation more useful for implementation decisions.

## Abbreviations

VR: virtual reality
XR: extended reality
